# Grafting Polytetrafluoroethylene Micropowder via in Situ Electron Beam Irradiation-Induced Polymerization

**DOI:** 10.3390/polym10050503

**Published:** 2018-05-06

**Authors:** Hui Wang, Yingfeng Wen, Haiyan Peng, Chengfu Zheng, Yuesheng Li, Sheng Wang, Shaofa Sun, Xiaolin Xie, Xingping Zhou

**Affiliations:** 1 Key Laboratory for Material Chemistry of Energy Conversion and Storage, Ministry of Education, School of Chemistry and Chemical Engineering, Huazhong University of Science and Technology, Wuhan 430074, China; whitley.wang@outlook.com (H.W.); wenyf.chem@foxmail.com (Y.W.); hypeng@mail.hust.edu.cn (H.P.); xlxie@mail.hust.edu.cn (X.X.); 2 National Anti-counterfeit Engineering Research Center, Huazhong University of Science and Technology, Wuhan 430074, China; zhengchengfu320@sohu.com; 3 School of Nuclear and Chemistry & Biology, Hubei University of Science and Technology, Xianning 437100, China; frank78929@163.com (Y.L.); ws1203@163.com (S.W.)

**Keywords:** electron beam irradiation-induced grafting, polytetrafluoroethylene, methyl methacrylate, interfacial interaction

## Abstract

Decreasing the surface energy of polyacrylate-based materials is important especially in embossed holography, but current solutions typically involve high-cost synthesis or encounter compatibility problems. Herein, we utilize the grafting of polytetrafluoroethylene (PTFE) micropowder with poly (methyl methacrylate) (PMMA). The grafting reaction is implemented via in situ electron beam irradiation-induced polymerization in the presence of fluorinated surfactants, generating PMMA grafted PTFE micropowder (PMMA–g–PTFE). The optimal degree of grafting (DG) is 17.8%. With the incorporation of PMMA–g–PTFE, the interfacial interaction between polyacrylate and PTFE is greatly improved, giving rise to uniform polyacrylate/PMMA–g–PTFE composites with a low surface energy. For instance, the loading content of PMMA–g–PTFE in polyacrylate is up to 16 wt %, leading to an increase of more than 20 degrees in the water contact angle compared to the pristine sample. This research paves a way to generate new polyacrylate-based films for embossed holography.

## 1. Introduction

Currently, fake and shoddy products are regarded as the second largest public nuisance after the drug trade in the world; they endanger the development of the economy and people’s health. In the face of this problem, anti-counterfeiting techniques need to provide more reliable solutions. To this end, many common anti-counterfeiting methods such as holography, bar coding, magnetic recording, and radio frequency identification (RFID) are employed [1,2,3,4]. Techniques involving optical manipulation have attracted considerable attention because of their optical features readily recognizable with the naked eye without the need for special equipment [5,6]. For instance, holograms [7], since the seminal work of Gabor in 1948 [8], have taken the top choice in daily optical anti-forgery owing to their angle-dependent blazing effect or three-dimensional dynamic images, visible to the naked-eye [9]. Embossed holograms, which can be easily reproduced on an industrial scale by transferring holographic images from a hot metal master plate to flexible polymer films under a press, are particularly important and have been increasingly applied for security in certificates, currency, bank cards, and other valuable goods.

Thermoplastic polyacrylate is commonly used for recording the transferred embossed holograms thanks to its outstanding transparency, good weather resistance, and high flexibility [10]. Nickel plate is typically employed as the master plate because of its ease of use in hologram production and relatively low cost. During the embossing process, holograms on the surface of a nickel master plate are transferred to the softened area of a polyacrylate film under pressure. The embossed parts on the polyacrylate film can be softened and reshaped through glass transition by heating to 160–180 ˚C [11]. The problem is that the softened polyacrylate always displays strong adhesion to the hot nickel plate. Consequently, it is hard to separate the embossed polyacrylate with the nickel master without reducing the quality of the embossed holograms [12].

To avoid strong adhesion of the polyacrylate to the hot metal plate, surface modification of the metal plate with an anti-sticking agent [13] and reducing the surface energy of the polyacrylate via copolymerization or blending [14,15,16] have been employed. To do this, fluoropolymers are considered to be the best materials to produce anti-adhesive performance owing to their excellent hydrophobic and oleophobic properties [17,18,19]. Their low surface energy and outstanding inertness primarily arise from the molecule structure. Because of the high electro-negativity and large atomic radius compared to hydrogen atoms, fluorine atoms in fluoropolymers exhibit a strong intramolecular repulsion force and thus arrange spirally along the carbon backbone as a protective sheath [20]. Moreover, high bond energy and low polarizability of C–F bonds give rise to weak intermolecular actions with fluoropolymer themselves or other materials [21]. As expected, the fully fluorinated polymer, i.e., polytetrafluoroethylene (PTFE), shows an extremely low surface energy (γ_s_ = 2.02 × 10^−2^ N/m) [22]. Nevertheless, the low surface energy of PTFE leads to inherently poor adhesion and compatibility with other materials [23,24]. To improve the compatibility of PTFE with polyacrylates, surface modification including chemical etching and high-energy irradiation is a good solution. Some fluorine atoms are removed from the PTFE surface, simultaneously generating new hydroxyl, carboxyl, and other functional groups [25]. The new grafted polymer surface could enhance adhesion of the fluoropolymers to other materials. For example, by grafting glycidyl methacrylate, the T-peel adhesion strength between PTFE film and aluminum foil increases significantly [26].

Among the techniques used to modify PTFE, high-energy irradiation such as plasma, ion beam, gamma ray, X ray, vacuum ultraviolet, and electron beam, are efficient and controllable [27,28,29,30,31,32]. No initiator or catalyst is required and a large-scale production without affecting the bulk properties can be achieved for meeting the criteria of practical applications. Particularly, electron beam irradiation has been well recognized to be advantageous for its high dose rate, uniform beam energy, less wettability loss over time, and large penetration depth [33,34]. Since the original research of electron beam irradiation-induced grafting on PTFE by Chapiro [35,36], pre-irradiation-induced grafting and in situ irradiation-induced grafting have been developed. In the former method, PTFE is firstly exposed to electron beam irradiation to produce trapped radicals or peroxy/hydroperoxy radicals, and then the radicals initiate monomers to polymerize on the PTFE surface [37,38]. In the latter method, grafting polymerization is carried out through irradiating both PTFE and monomers simultaneously [39,40]. Compared with pre-irradiation-induced grafting reaction, the in situ irradiation-induced grafting polymerization gives a higher degree of grafting (DG) because of a higher radical yield and less radical loss.

Various vinyl monomers such as acrylic acid, styrene, N-isopropylacrylamide, and 4-styrenesulfonate can be grafted onto PTFE [29,41]. A water contact angle decrease on the PTFE surface is an evidence of successful grafting [39]. The modified PTFE has also been widely applied to adsorbent materials [42], proton exchange membranes [38], and biomedical materials [43]. Despite extensive work on the grafting process, the compatibility improvement of PTFE with other polymer matrices remains a challenge [44,45]. Severe aggregation usually occurs which is hard to avoid even when employing large irradiation dosage or monomer concentration during grafting [46,47,48]. Thus, in order to meet the requirements of embossed holograms, the optimization of DG and PTFE loading is highly anticipated.

In this work, in situ irradiation-induced grafting polymerization of methyl methacrylate (MMA) onto PTFE micropowder was performed, generating poly (methyl methacrylate) (PMMA) grafted PTFE micropowder (PMMA–g–PTFE). The PMMA–g–PTFE was then incorporated into a polyacrylate matrix to fabricate composite films. To achieve better dispersion of the micropowder and to reduce aggregation, we employed fluorosurfactant to disperse the micropowder into a monomer solution prior to electron beam irradiation [49]. Optimization of DG and PMMA–g–PTFE loading in composite films was exerted to decrease the surface energy of the polyacrylate-based composites while maintaining a good interfacial action. The results pave the way for generating new polyacrylate-based films for use in embossed holography.

## 2. Materials and Methods

### 2.1. Materials

Commercially available PTFE micropowder (Dyneon™ TF-9205, 3M, Neuss, Germany) and fluorosurfactant ((C_2_H_4_O)_n_(CF_2_CF_2_)_m_F Zonyl™ FSN-100, Dupont, Wilmington, DE, USA) were used as received. Methyl methacrylate (MMA, purity: 99%), butyl acetate (purity: 99%), toluene (purity: 99.5%), butanone (purity: 99.5%), acetone (purity: 99.5%), Mohr’s salt (FeH_8_N_2_O_8_S_2_·6H_2_O) were purchased from Sinopharm Chemical Reagent Co., Ltd., Shanghai, China and utilized directly without any further purification. Polyethylene terephthalate (PET) film and polyacrylate resin (50% solid content) were supplied by Huagong Image Technology Development Co., Ltd., Wuhan, China. Ultrapure water with a conductivity of 18.3 S/cm was used as the grafting medium.

### 2.2. Grafting PTFE Micropowder with PMMA

Grafting PTFE micropowder with PMMA was implemented through in situ electron beam irradiation-induced polymerization of MMA. Prior to grafting polymerization, 3 g PTFE micropowder, 3 mL MMA, Mohr’s salt, and fluorosurfactant were mixed and diluted with ultrapure water. The contents of Mohr’s salt and fluorosurfactant were controlled to be 0.3 wt % and 0.05 wt % of the amount of MMA, respectively. To dial the DG, the MMA concentration in the mixture was controlled to be 3, 6, 9, 12, and 15 wt %, respectively, by controlling the amount of added water. Emulsions were obtained through shearing the oil–water mixture for 5 min at a speed of 8000 r/min. The resulting emulsion was then degassed in a thin polyethylene bag to remove air and finally sealed. The grafting reaction was initiated at room temperature by irradiating the samples with accelerated electrons under an Electron Beam Processing System (Wasik Associates Inc., Dracut, MA, USA). The electron energy and beam current were set as 1.0 MeV and 18 mA, respectively. The irradiation dose was controlled to be 20, 40, 60, and 80 kGy, separately, at a dose rate of 20 kGy/pass. Subsequent to the irradiation-induced grafting reaction, the PMMA–g–PTFE micropowder was centrifugalized for 6 min at a speed of 8000 r/min. To remove the residual monomer and homopolymer formed during irradiation, the centrifuged PMMA–g–PTFE micropowder was first washed with deionized water and then Sohxlet-extracted with acetone for 72 h at 75 °C until a constant weight was achieved. Finally, the PMMA–g–PTFE micropowder was dried at 65 °C in vacuum until a constant weight was achieved.

### 2.3. Formulation of Polyacrylate/PMMA–g–PTFE Composite Films

Commercially available polyacrylate resin in 2 g was first dissolved in a mixed solvent composed of 2.8 mL of toluene and 1.9 mL of butanone. Then, the PMMA–g–PTFE micropowder in a predesigned amount was dispersed in 2.8 mL of butyl acetate, followed by ultrasonication for 30 min. Subsequently, the individual PMMA–g–PTFE suspension and polyacrylate resin were mixed by ultrasonication for 1 h. To form composite films, 200 μL of resulting mixture was spin-cast onto PET films (24 mm length × 24 mm width), and then dried at 120 °C for 2 min. The content of PMMA–g–PTFE in these composite films was controlled to be 2, 4, 6, 8, 10, 12, 14, and 16 wt %, respectively, relative to polyacrylate. As a control, neat polyacrylate films and polyacrylate/PTFE composite films with the same micropowder contents were also fabricated following the aforementioned steps.

### 2.4. Characterization

Fourier transform infrared spectroscopy (FTIR, Equinox 55, Bruker, Karlsruhe, Germany) was used to identify the functional group. KBr pellets were employed to support the samples during characterization. Data were collected in the transmission mode after cumulating 32 scans with a resolution of 2 cm^−1^.

Thermogravimetric analysis (TGA, 4000, Perkin Elmer, Waltham, MA, USA) was performed under nitrogen atmosphere from 30 to 700 °C at a ramp rate of 10 °C/min.

X-ray photoelectron spectroscopy (XPS, Axis Ultra DLD, Kratos, Manchester, UK) was used to analyze the chemical bonding in the PMMA–g–PTFE and PTFE micropowder. Photoemission was excited by a monochromatic Al-K_α_ radiation. Survey scans were carried out from 1200 to 0 eV with an interval of 1.0 eV. Narrow scans were exerted with 0.05 eV steps. All XPS spectra were charge-compensated to C1s at 285 eV. Linear baseline for background subtraction and Gaussian-Lorentzian function were used for peak fitting.

The morphology of the pristine PTFE, PMMA–g–PTFE, and their composite films with polyacrylate were studied using field-emission scanning electron microscopy (FE-SEM, Sirion 200, FEI, Hillsboro, OR, USA) at an accelerating voltage of 10 kV. PTFE and PMMA–g–PTFE micropowders for SEM characterization were prepared by placing a drop of the acetone-suspended micropowder onto a silicon wafer and dried in an ambient atmosphere. The composite films for SEM characterization were prepared by placing the polyacrylate/micropowder suspension onto PET films and dried at 120 °C. A duration of 5 min was chosen to fully remove the solvent prior to SEM characterization. Composite films were peeled off from the PET substrate when conducting the SEM characterization to avoid the background signal from PET. All samples were coated with platinum on the top surface before the test.

The dispersion stability of the polyacrylate/PMMA–g–PTFE mixture and the polyacrylate/PTFE mixture were studied by ultraviolet-visible spectrophotometer (UV-vis, UV 2600, Shimadzu, Kyoto, Japan) at a wavelength of 600 nm. Data were collected in the transmission mode at a rate of 10 scan/min over the course of 60 min.

Water contact angles were measured by the static contact angle test (CA, JC2000C1, Zhongchen Powereach Company, Shanghai, China). Around 0.5 μL of deionized water was dropped on the sample surface at room temperature. For each sample, ten independent tests on different surface parts were exerted to give mean values with standard deviations.

Atomic force microscopy (AFM, SPM 9700, Shimadzu, Kyoto, Japan) was conducted on the surface of composite films in the tapping mode with a resonant frequency of 300 kHz. The root-mean-square-roughness (RMS) was calculated from the roughness profile determined by AFM.

## 3. Results and Discussion

### 3.1. Determination of DG in PMMA–g–PTFE micropowder

**Figure 1:**
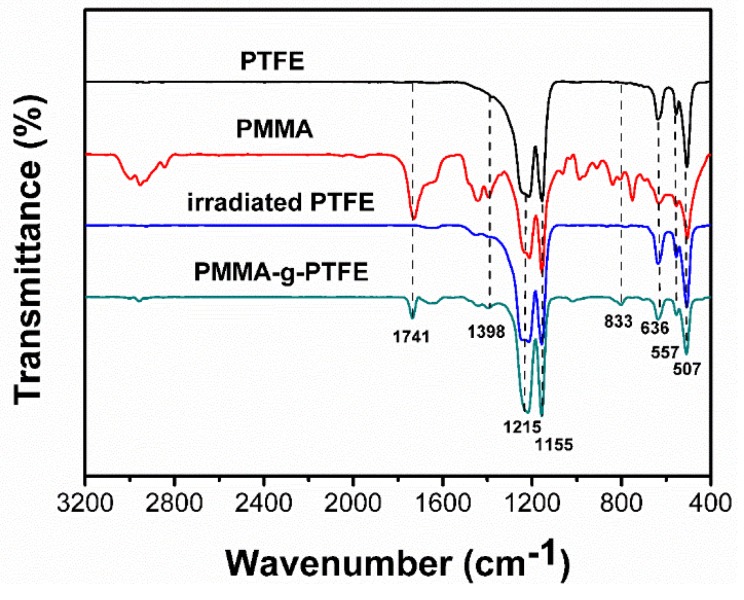
Fourier transform infrared spectroscopy (FTIR) spectra of the pristine polytetrafluoroethylene (PTFE), poly (methyl methacrylate) (PMMA), irradiated PTFE and PMMA grafted PTFE micropowder (PMMA–g–PTFE) formed under a dosage of 80 kGy.

Initially, to confirm the successful grafting reaction of PMMA to PTFE, FTIR spectra of the pristine PTFE and PMMA–g–PTFE were captured, individually. As illustrated in Figure 1, both pristine PTFE and PMMA–g–PTFE display the clear characteristic bands at 1155 and 1215 cm^−1^, because of the symmetrical stretching and asymmetrical stretching of CF_2_, respectively. The peaks at 507-636 cm^−1^ are ascribed to CF_2_ rocking, wagging, and bending vibrations. The spectrum of PMMA–g–PTFE micropowder shows an absorption at 1741 cm^−1^, which is associated with C=O stretching vibration of the ester group in grafted PMMA. This absorption is also clear for the PMMA homopolymer, while is absent in PTFE. In addition, peaks appear at 1398 and 833 cm^−1^ arising from CH_3_ on the grafted PMMA chain [50]. To exclude the possible formation of carbonyl and hydroxyl groups on PTFE under irradiation, the pristine PTFE micropowder was directly irradiated at the same dose in the absence of monomers. Results show that there is no carbonyl or hydroxyl group absorption in the irradiated PTFE. These FTIR results imply the possible chemical coupling of PTFE with grafted PMMA in the PMMA–g–PTFE micropowder.

**Figure 2:**
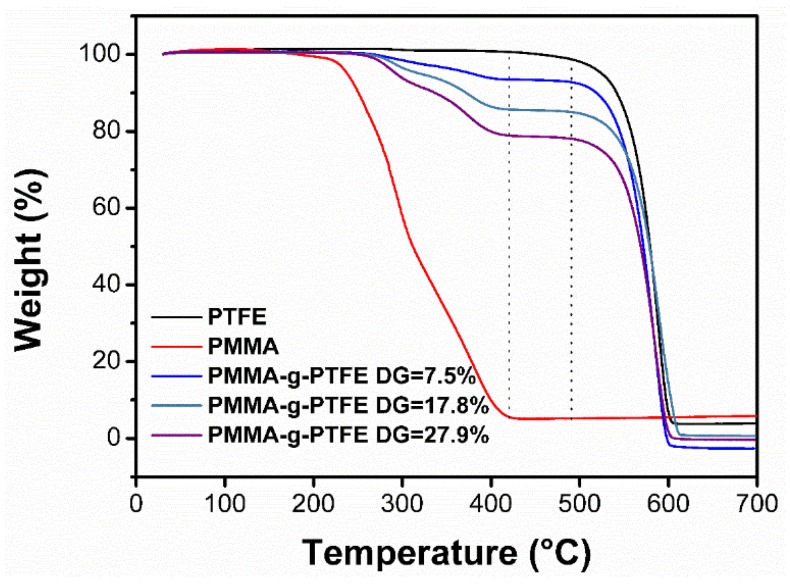
Thermogravimetric analysis (TGA) curves of the pristine PTFE, PMMA, PMMA–g–PTFE with varied degree of grafting (DG) of 7.5%, 17.8%, and 27.9%, respectively.

DG is simply the mass ratio of grafted PMMA to PTFE. To determine the DG in PMMA–g–PTFE, TGA was exerted. As shown in Figure 2, the degradation temperature range of pristine PTFE is 490–610 °C while that for PMMA is 180–420 °C, in good agreement with previous reports [51,52]. It is clear that the degradation of PMMA and PTFE are independent from each other. Thus, for the irradiation-generated PMMA–g–PTFE micropowder, the DG of PMMA can be calculated from the weight loss at two distinct degradation stages,

(1) $${\mathrm{DG}=[(m}_{f}-m_{i}{)/m}_{i}]\times 100\%$$

where m*_f_* and m_i_ signify the mass of PMMA–g–PTFE micropowder and PTFE micropowder core, respectively.

**Figure 3:**
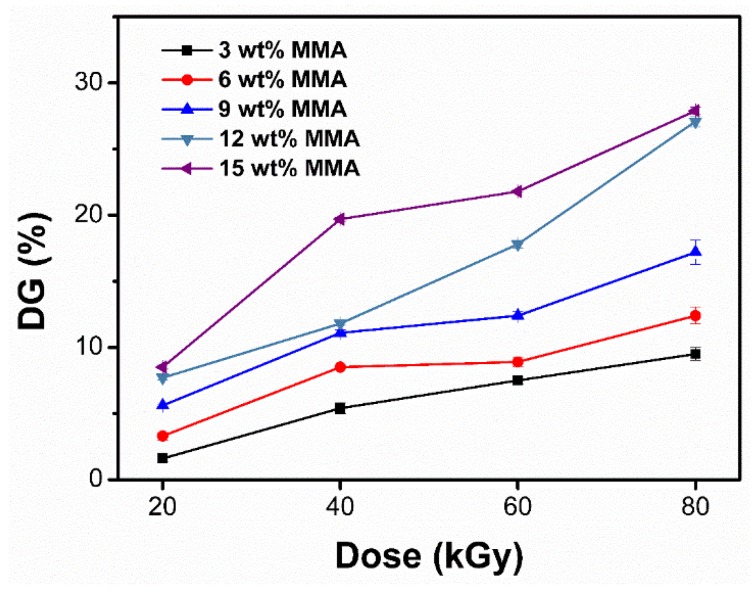
Overall DG for the PMMA–g–PTFE micropowder formed at different methyl methacrylate (MMA) concentrations and under varied doses.

The DG in PMMA–g–PTFE can be dialed by varying the initial monomer concentration and irradiation dose. As depicted in Figure 3, the DG is found to increase with monomer concentration and irradiation dose. It is worth noting that approximately twice the DG is achieved when increasing the irradiation dose from 20 to 40 kGy. Further increasing the irradiation dose gives less DG increments. Radical concentration and monomer diffusion are considered to be the two main reasons. The grafting reaction is expected to happen as follows. When exposed to electron beams, the PTFE main chain cleavage occurs to generate free radicals. The carbon center radicals are believed to contribute to the grafting polymerization via initiating the surrounded monomers to polymerize. Thus, it is easy to understand that a higher concentration of radicals or monomers would accelerate the grafting polymerization, in accordance with previous reports [53]. Meanwhile, the liquid MMA is a good solvent for PMMA, thus providing better mobility for the growing PMMA chains which leads to higher propagation efficiency. Although increasing the dose can increase the DG, a higher dose may also lead to more bimolecular termination that consequently causes crosslinked polymers which are not soluble in any solvent. As a consequence, the DG increment may be impeded [54].

In addition, the sample temperature was noted to rise rapidly at a high irradiation dose, although it is hard to measure this precisely. The temperature increase is also expected to accelerate grafting reaction through improving the MMA molecular diffusion [55].

**Figure 4:**
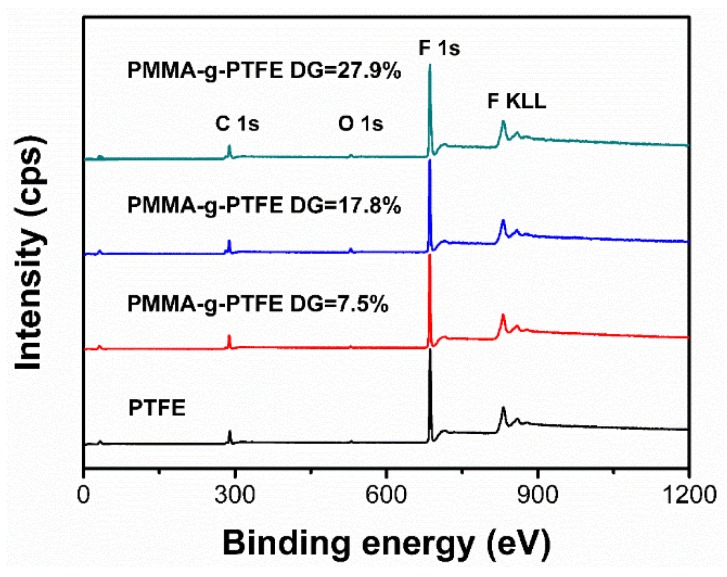
X-ray photoelectron spectroscopy (XPS) survey scans of the pristine PTFE and PMMA–g–PTFE micropowder with an individual DG of 7.5%, 17.8%, and 27.9%.

To further verify the chemical bonding between PTFE and PMMA in the PMMA–g–PTFE micropowder, XPS surveys of the pristine PTFE and PMMA–g–PTFE micropowder were exerted. For instance, the XPS data for the micropowder with DG of 7.5%, 17.8% and 27.9%, respectively, are presented in Figure 4. For the pristine PTFE micropowder, C1s core level at 290 eV and F1s core level at 686 eV assigned to the C–F bond are clear. By contrast, the carbon doublet peaks at 285.0 eV and 292.1 eV are observed in the PMMA–g–PTFE micropowder, while the pristine PTFE survey has only a single carbon peak.

**Figure 5:**
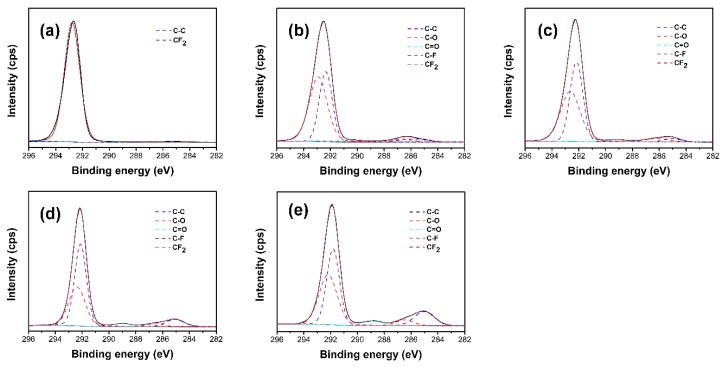
The XPS C1s narrow scan spectra of (**a**) the pristine PTFE; and (**b**–**e**) PMMA–g–PTFE micropowder formed under varied doses of (**b**) 20 kGy; (**c**) 40 kGy; (**d**) 60 kGy; and (**e**) 80 kGy, respectively. The DG for each is listed in Table 1.

To get deeper insights into the chemical bonding of PMMA with PTFE in the PMMA–g–PTFE, XPS C1s narrow scans were analyzed. Here, to make the comparison much clearer, we focused on the PMMA–g–PTFE formed from the oil-in-water emulsion with 3 wt % MMA. The irradiation dose was varied from 20 to 80 kGy to tailor to the DG value, as discussed in Figure 3. As shown in Figure 5a, the ungrafted PTFE micropowder exhibits only one peak at 292.5 eV attributing to CF_2_ bonds in the repeating unit of -(CF_2_–CF_2_)-. By contrast, for the PMMA–g–PTFE micropowder, the C1s core level exhibits broad peaks centered at 285.0 eV and 292.1 eV, respectively, as illustrated in Figure 5b–e, which can be deconvoluted to several peaks associated to C–C (284.9 eV), C–O (286.2 eV), C=O (288.9 eV), C–F (291.9 eV), and CF_2_ bonds (292.5 eV), respectively [56,57]. The peaks assigned to C–C (284.9 eV), and C=O (288.9 eV) bonds become clearer when the irradiation dosage increases (Figure 5b–e). The atomic ratios and bond proportions calculated from the XPS C1s narrow scans are listed in Table 1. The F/C atomic ratio of pristine PTFE is 1.82, in agreement with the theoretical value. A small amount of oxygen (atomic concentration of 0.45%) in the pristine PTFE micropowder might be caused by H_2_O or oxygen from air [58]. The concentrations of C–C, C–O, C=O, and C–F bonds clearly increase in the PMMA–g–PTFE, in comparison with the pristine PTFE, and increase with the augmentation of DG in the PMMA–g–PTFE. Whereas, the CF_2_ content decreases significantly. As a result, the F/C atomic ratio in the PMMA–g–PTFE micropowder drops from 1.69 to 1.52 when increasing the DG from 1.4% to 9.8%, while the O/C atomic ratio increases from 0.01 to 0.05. It should be pointed out that the increase in C–F content with the augmentation of irradiation dosage indicates the chemical attachment of PMMA to PTFE through the CF_2_ bonds breaking.

**Figure 6:**
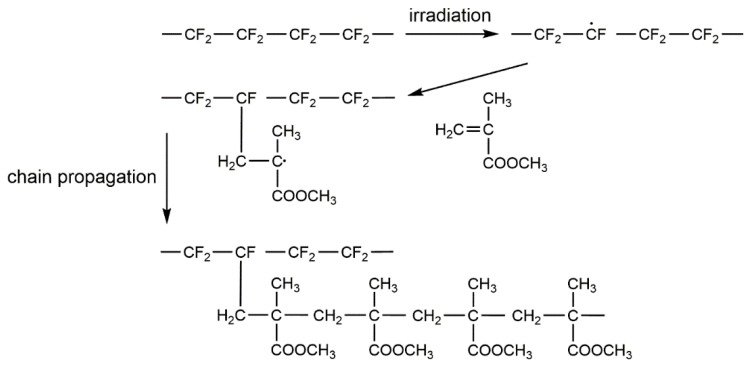
In situ irradiation-induced grafting polymerization mechanism of PMMA–g–PTFE micropowder.

The generated ∙CF- active sites after destroying the CF_2_ bond are well accepted to simultaneously initiate the grafting polymerization (Figure 6) [53]. The breaking of hydrophobic CF_2_ group and formation of hydrophilic groups, such as C=O, are able to increase the surface energy and wettability of PMMA–g–PTFE micropowder. To prove this, a water contact angle investigation on micropowder tablets was implemented. These tablets were made from micropowder under a pressure of 200 MPa. As illustrated in Figure S1, grafting PMMA chains on the surface of PTFE micropowder gives rise to a decrease in the water contact angle from 101.8 ± 1.9° to 96.7 ± 1.0°, implying an increase in hydrophilicity. The grafting modification of PTFE with PMMA is also believed to increase the miscibility between PTFE and the polyacrylate matrix as the solubility parameter imbalance decreases.

It is well known that the glass transition temperature, T_g_, of polymer is mainly related to the segmental chain mobility and free volume. The grafted PMMA chains on PTFE exhibit a higher T_g_ of 121 °C than that of the PMMA homopolymer (103 °C). The primary reason for raising the T_g_ is the limited segmental chain mobility of grafted PMMA. Meanwhile, the strong polarity of C–F bonds leads to larger dipole–dipole forces between the PTFE and PMMA molecules [59]. Lastly, because of the aggregation of PMMA–g–PTFE micropowder, the free volume of PMMA chains decreases with an increase of DG [60], thus leading to constrained segmental mobility.

### 3.2. Micromorphology of PMMA–g–PTFE Micropowder

**Figure 7:**
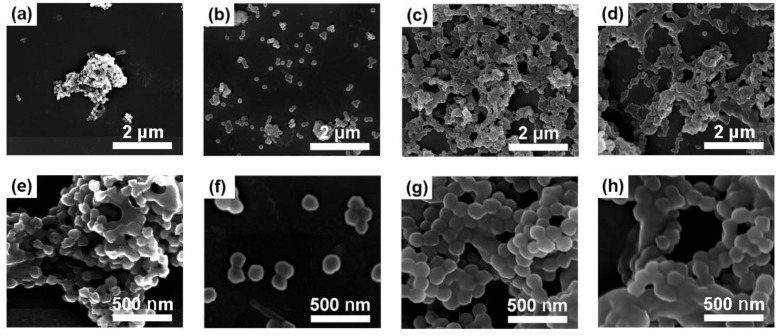
SEM images of (**a**,**e**) the pristine PTFE, and PMMA–g–PTFE micropowder with a DG of (**b**,**f**) 7.5%; (**c**,**g**) 17.8%; and (**d**,**h**) 27.9%, respectively.

Crosslinking between different micropowders could cause aggregation, that is, clustering. For the pristine PTFE micropowder, irregular shapes and severe aggregation are clear under SEM (Figure 7a,e), mainly because of its hydrophobic and oleophobic nature and poor dispersity in solvents. After electron beam irradiation, smaller PMMA–g–PTFE micropowders with a relatively narrow size distribution (from 130 to 160 nm) are given; this is because of the grafted PMMA chains on the PTFE surface (Figure 7b,f). Nevertheless, crosslinking probably occurs between PMMA chains on the surface of PTFE particles, especially under a high dose of irradiation, which results in clusters (Figure 7c,d,g,h). Less clusters with a lower DG indicates that the crosslinking primarily occurs between PMMA chains on different PTFE particles.

It should be noted that the addition of fluorosurfactant greatly enhanced the dispersion of pristine PTFE micropowder in the monomer solution (Figure S2). Without fluorosurfactant, crosslinking between PMMA on the surface of different particles is significant and hard to extract (Figure S3).

### 3.3. Polyacrylate/PMMA–g–PTFE Composite Films

**Figure 8:**
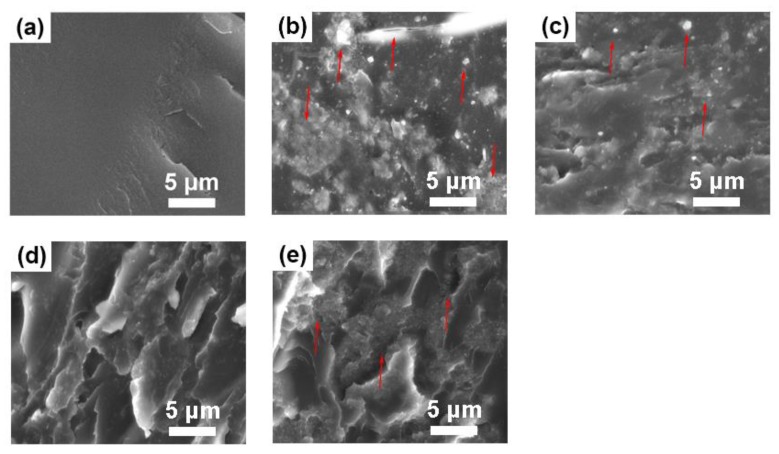
SEM images of fracture surfaces of (**a**) the pristine polyacrylate film; (**b**) polyacrylate composite film with 10 wt % of untreated PTFE; and (**c**–**e**) polyacrylate composite film with 10 wt % of PMMA–g–PTFE. The DG in the PMMA–g–PTFE was controlled to be (**c**) 7.5%; (**d**) 17.8%; and (**e**) 27.9%, respectively.

Polyacrylate/PMMA–g–PTFE composites were formed by dispersing the PMMA–g–PTFE micropowder into the commercially available polyacrylate resin. To make a clear comparison, the PMMA–g–PTFE micropowders with DG of 7.5%, 17.8%, and 27.9% were investigated, respectively. To understand the dispersity of PMMA–g–PTFE in these polyacrylate, fracture surfaces of the pristine polyacrylate film, polyacrylate composites with untreated PTFE and polyacrylate/PMMA–g–PTFE composite films with 10 wt % micropowder were evaluated using SEM. As displayed in Figure 8a, a smooth surface for the polyacrylate is clear. When doped with untreated PTFE, aggregates showing a poor dispersity in polyacrylate are clear (Figure 8b). Poor interfacial action between PTFE and polyacrylate is clear from the cracks as highlighted by the red arrows [27]. In contrast, the polyacrylate/PMMA–g–PTFE composite films exhibit a much better interfacial action (Figure 8c–e), because of the close solubility parameters between grafted PMMA and polyacrylate. The solubility of PMMA was calculated to be 18.6 (MJ/m^3^)^1/2^ while that for polyacrylate was estimated to be 17.8 (MJ/m^3^)^1/2^ from the mixed solvent. No significant aggregated PMMA–g–PTFE clusters were shown when the DG equals 7.5%. Yet, such low degree of grafting provides insufficient interfacial action between PTFE and polyacrylate, thus small white domains of PTFE are clear. Further increasing the DG gives rise to better interfacial action and good dispersity. The PMMA–g–PTFE with a DG of 17.8% results in the best dispersion and interfacial adhesion between PTFE with polyacrylate. Compared with PMMA–g–PTFE micropowder with a low DG, PMMA–g–PTFE micropowder with 27.9% DG prefers to aggregate as large clusters in polyacrylate.

**Figure 9:**
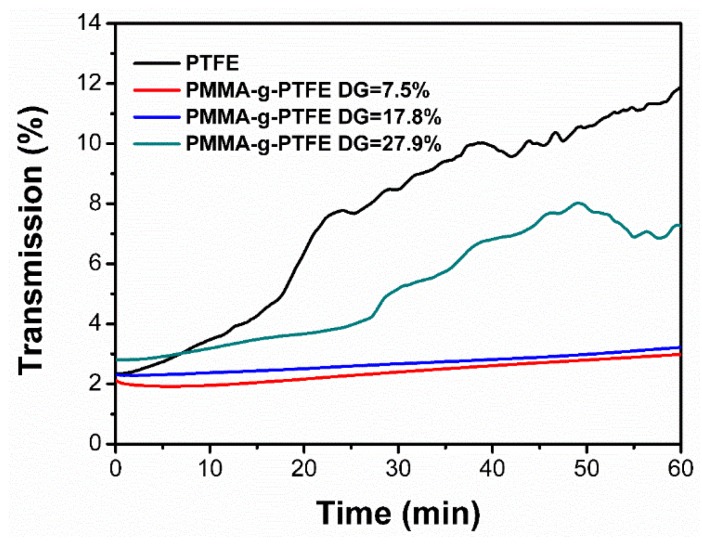
Transmission at 600 nm of polyacrylate solution when adding 10 wt % of untreated PTFE, and PMMA–g–PTFE with a DG of 7.5%, 17.8%, and 27.9%, respectively.

To better understand the improved interfacial adhesion between PMMA–g–PTFE micropowder and the polyacrylate matrix, the dispersion stability of grafted PTFE with a DG of 7.5%, 17.8%, and 27.9%, respectively, were compared with the pristine PTFE (Figure 9) [61,62]. For all polyacrylate/PMMA–g–PTFE and polyacrylate/PTFE mixtures in solution, close initial transmission intensities are shown, indicating a similar homogeneity. After being left to stand for around 10 min, the transmission intensity gradually increases because of the micropowder precipitation. Clearly, the transmission intensities of the polyacrylate/PMMA–g–PTFE mixture with different DG values are always lower than that of the polyacrylate/PTFE mixture. The untreated PTFE micropowder is almost completely precipitated after being stood for 2 h (Figure S4). It is worth pointing out that the transmission intensity of the polyacrylate/PMMA–g–PTFE mixture with 27.9% DG increases much faster than that of mixtures with a lower DG. It means that the PMMA–g–PTFE with a DG of 27.9% tends to precipitate more readily because of the formed aggregation, as aforementioned.

### 3.4. Surface Properties of the Polyacrylate/PMMA–g–PTFE Composite Films

It is commonly accepted that a material with a higher solid surface energy will have a better wettability with a given liquid. According to the Young–Dupré equation, a decrease in the contact angle indicates an increase in the work of solid–liquid adhesion, namely better wettability [63]. Accordingly, a hydrophobic solid surface with lower surface energy always exhibits higher water contact angle. This can be used as an important measurement criterion of wettability.

**Figure 10:**
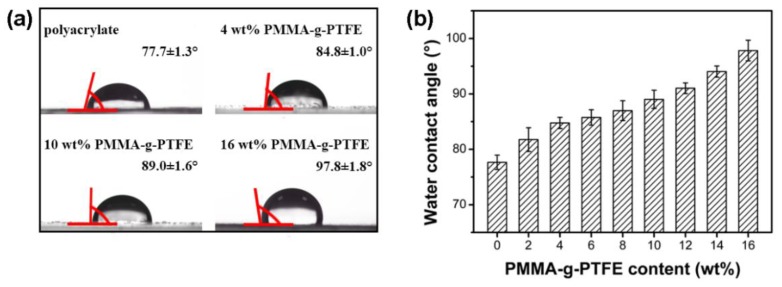
(**a**) Photographs of a water droplet on the top surface of composite films; and (**b**) water contact angle as a function of PMMA–g–PTFE micropowder content.

The following section focuses on the PMMA–g–PTFE with a DG of 17.8%. With incorporated PMMA–g–PTFE micropowder, the wettability of the polyacrylate composite films was studied by measuring the static water contact angle (Figure 10). As expected, the water contact angle increases with the augmentation of PMMA–g–PTFE. The water contact angle on the top surface of pristine polyacrylate film is 77.7°, while that of polyacrylate composite films increases by 26% to 97.8° when loaded with 16 wt % of PMMA–g–PTFE.

**Figure 11:**
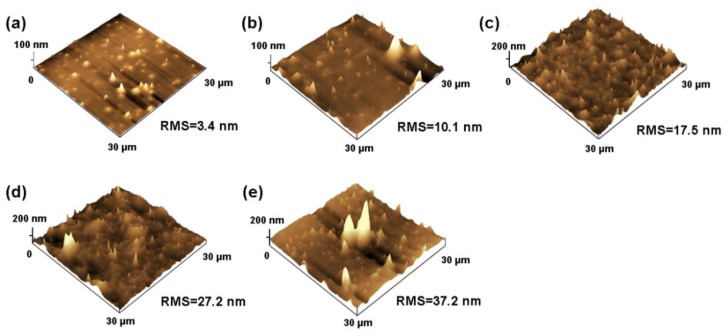
Surface morphology obtained from atomic force microscopy (AFM) for spin-coated composite films with (**a**) 4 wt %; (**b**) 10 wt %; (**c**) 12 wt %; (**d**) 14 wt %; and (**e**) 16 wt % PMMA–g–PTFE micropowder, respectively. The DG of PMMA–g–PTFE is 17.8% in all samples.

**Figure 12:**
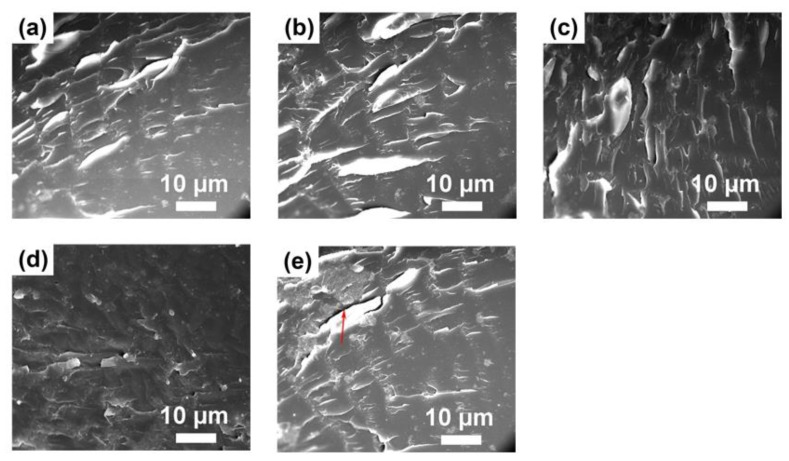
SEM images of fracture surfaces of composite films with (**a**) 4 wt %; (**b**) 10 wt %; (**c**) 12 wt %; (**d**) 14 wt %; and (**e**) 16 wt % of PMMA–g–PTFE micropowder, respectively. The DG of PMMA–g–PTFE is 17.8% in all samples.

It has been known that the surface contact angle is related to the chemical composition and surface morphology [64]. To further explain the water contact angle results, the surface roughness of polyacrylate/PMMA–g–PTFE composite films was investigated using AFM (Figure 11). The composite films display “humps” in different sizes composed of aggregated PMMA–g–PTFE micropowder clusters. The roughness (RMS) of the composite film is found to increase from 3.4 to 37.2 nm with an augmentation of PMMA–g–PTFE from 4 wt % to 16 wt %. For the composite film with 4 wt % PMMA–g–PTFE, the size of aggregated PMMA–g–PTFE clusters on the surface is clearly smaller than that of the films with higher PMMA–g–PTFE contents. The density of micropowder on the film surface increases significantly at a high PMMA–g–PTFE loading. These facts are consistent with those observed in SEM of fracture surfaces of polyacrylate/PMMA–g–PTFE composite films (Figure 12). The PMMA–g–PTFE micropowder tended to aggregate at a loading of 16 wt %, which resulted in large aggregated PMMA–g–PTFE clusters and rough surfaces like the “lunar surface”. With micropowder loadings of 12 wt % and 14 wt %, the PMMA–g–PTFE disperse homogeneously in polyacrylate and the raising surface roughness contributes to a large water contact angle to some extent. Thus, the optimization of both DG and loading of PMMA–g–PTFE are important to increase the interfacial action between PTFE and polyacrylate and also to dial the surface property of polyacrylate.

## 4. Conclusions

PMMA grafted PTFE, referred to as PMMA–g–PTFE, was successfully prepared via in situ electron beam irradiation-induced radical polymerization of MMA on the PTFE surface. The formation of oil-in-water emulsion using a fluorosurfactant was found to be critical during the grafting reaction. The chemical bonding of PMMA to PTFE was identified by FTIR and XPS. The degree of grafting, DG, was readily dialed by tuning the irradiation dosages and MMA concentration in the initial emulsion, and easily determined by TGA. The PMMA–g–PTFE was then added into commercially available polyacrylate to decrease the surface energy. Optimization of both DG and PMMA–g–PTFE loading was recognized to be important to improve the uniformity of polyacrylate/PMMA–g–PTFE composite films. With a 14 wt % PMMA–g–PTFE loading, the micropowder shows the optimized dispersity in the polyacrylate matrix and surface energy. This finding paves a way to formulate new polymer composites for advanced applications in embossed holograms.

## Figures and Tables

**Table 1:** Atomic ratios and bond proportions of the pristine polytetrafluoroethylene (PTFE) and poly (methyl methacrylate) (PMMA) grafted PTFE micropowder (PMMA–g–PTFE) obtained from X-ray photoelectron spectroscopy (XPS)^1^.

Entry	Dose (kGy)	DG (%)	Atomic Ratio		Bond Proportion (%)				
			F/C	O/C	C–C	C–O	C=O	C–F	CF_2_
1	0	0	1.82	0.005	1.37	0	0	0	98.63
2	20	1.4	1.69	0.01	3.59	2.34	2.56	38.67	52.84
3	40	5.1	1.67	0.02	3.65	2.31	2.78	49.65	41.61
4	60	7.7	1.65	0.03	7.28	2.75	3.39	52.89	33.69
5	80	9.8	1.52	0.05	11.56	4.18	3.98	53.14	27.14

^1^ Entry 1: pristine PTFE micropowder; entry 2–5: PMMA–g–PTFE micropowder; The bond proportion and elemental ratio were calculated from the peak areas shown in Figure 5; degree of grafting (DG) values were determined from thermogravimetric analysis (TGA) and dialed by varying the irradiation dosages on the emulsion with 3 wt % of methyl methacrylate (MMA).

## Supplementary Materials

The following are available online at http://www.mdpi.com/2073-4360/10/5/503/s1, Figure S1: Photographs of a water droplet on the surface of (a) PTFE tablet and (b) PMMA–g–PTFE tablet with a DG of 27.9%; Figure S2: Photographs of suspensions of the pristine PTFE micropowder dispersed in MMA monomer solution (a) with 0.05 wt % fluorosurfactant and (b) without fluorosurfactant; Figure S3: SEM images of the PMMA–g–PTFE micropowder obtained with MMA monomer solution (a) with 0.05 wt % fluorosurfactant and (b) without fluorosurfactant; Figure S4: Photographs of suspensions of (a) the pristine PTFE and (b) PMMA–g–PTFE micropowder dispersed in polyacrylate solution after being stood for 2 h.
