# Supplementary material for: Grafting Polytetrafluoroethylene Micropowder via in Situ Electron Beam Irradiation-Induced Polymerization
Source: Polymers (Basel). 2018 May 6;10(5):503. doi: 10.3390/polym10050503 (PMC6415420; doi:10.3390/polym10050503)
Supplement: Supplementary file 1 [file polymers-10-00503-s001.pdf]

polymers

Supplementary materials: Grafting Polytetrafluoroethylene Micropowder via in Situ Electron Beam Irradiation-Induced Polymerization

Hui Wang [1], Yingfeng Wen [1], Haiyan Peng [1], Chengfu Zheng [2], Yuesheng Li [3], Sheng Wang [3], Shaofa Sun [3,*], Xiaolin Xie [1,2] and Xingping Zhou [1,*]

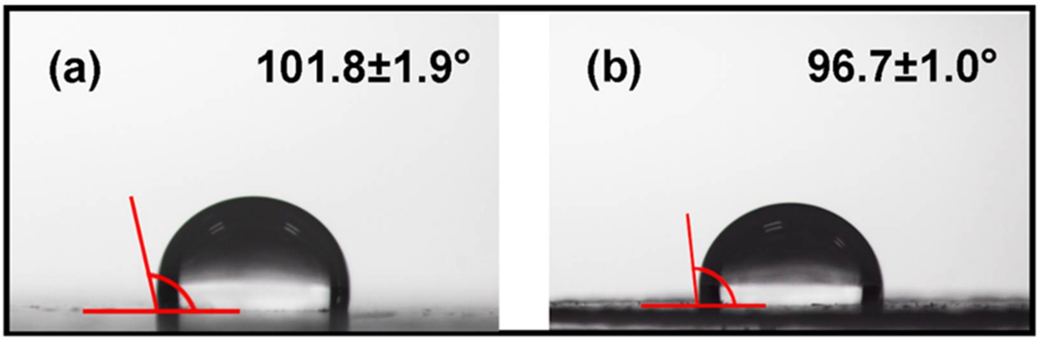

Figure S1. Photographs of a water droplet on the surface of (**a**) PTFE tablet and (**b**) PMMA-g-PTFE tablet with a DG of 27.9%.

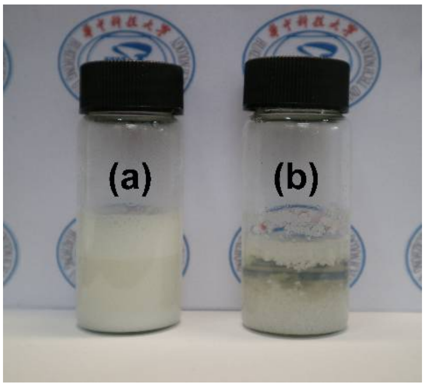

Figure S2. Photographs of suspensions of the pristine PTFE micropowder dispersed in MMA monomer solution (**a**) with 0.05 wt% fluorosurfactant and (**b**) without fluorosurfactant.

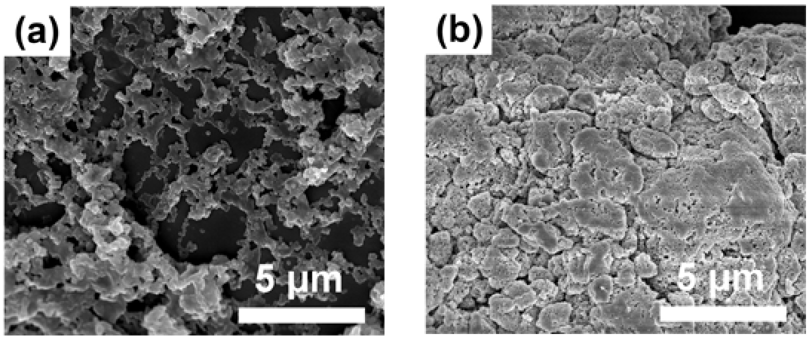

Figure S3. SEM images of the PMMA-g-PTFE micropowder obtained with MMA monomer solution (**a**) with 0.05 wt% fluorosurfactant and (**b**) without fluorosurfactant.

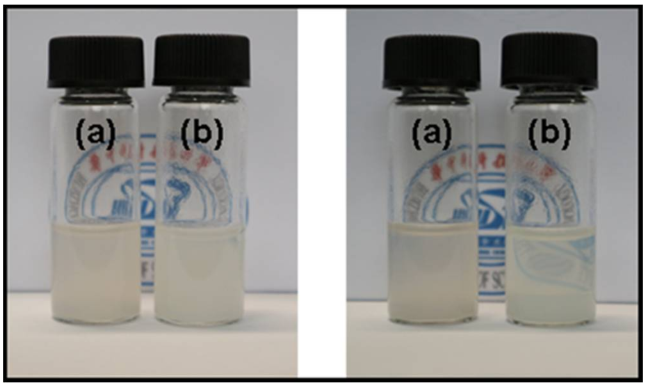

Figure S4. Photographs of suspensions of (**a**) the pristine PTFE and (**b**) PMMA-g-PTFE micropowder dispersed in polyacrylate solution after being stood for 2 h.
